# Mechanochemical regulations of RPA's binding to ssDNA

**DOI:** 10.1038/srep09296

**Published:** 2015-03-19

**Authors:** Jin Chen, Shimin Le, Anindita Basu, Walter J. Chazin, Jie Yan

**Affiliations:** 1 Mechanobiology Institute, National University of Singapore, 117411 Singapore; 2 Department of Physics, National University of Singapore, 117542 Singapore; 3 Departments of Biochemistry and Chemistry, Center for Structural Biology, Vanderbilt University, Nashville, TN 37232, USA; 4 Centre for Bioimaging Sciences, National University of Singapore, 117546 Singapore

## Abstract

Replication protein A (RPA) is a ubiquitous eukaryotic single-stranded DNA (ssDNA) binding protein that serves to protect ssDNA from degradation and annealing, and as a template for recruitment of many downstream factors in virtually all DNA transactions in cell. During many of these transactions, DNA is tethered and is likely subject to force. Previous studies of RPA's binding behavior on ssDNA were conducted in the absence of force; therefore the RPA-ssDNA conformations regulated by force remain unclear. Here, using a combination of atomic force microscopy imaging and mechanical manipulation of single ssDNA tethers, we show that force mediates a switch of the RPA bound ssDNA from amorphous aggregation to a much more regular extended conformation. Further, we found an interesting non-monotonic dependence of the binding affinity on monovalent salt concentration in the presence of force. In addition, we discovered that zinc in micromolar concentrations drives ssDNA to a unique, highly stiff and more compact state. These results provide new mechanochemical insights into the influences and the mechanisms of action of RPA on large single ssDNA.

Replication protein A (RPA) is the primary eukaryotic single-stranded DNA (ssDNA) binding protein that has critical roles in a range of DNA transactions including DNA replication, recombination and repair^1^,^2^,^3^,^4^. RPA's role during these processes is to protect and organize ssDNA, which is prone to be degraded by endonucleases and other factors^5^,^6^,^7^. Further, it plays a central role as a target for many downstream factors involved in these transactions^8^,^9^. RPA is a prototypical modular protein with multiple domains connected by flexible linkers. The RPA heterotrimer consists of RPA70, RPA32 and RPA14 subunits, which are organized into four independent structural modules: the RPA70C/32D/14 trimer core, the RPA70AB DNA binding module, and the two protein recruitment domains RPA70N and RPA32C^10^,^11^,^12^,^13^.

RPA binds ssDNA with a 5′ to 3′ polarity arising from the ordered binding of ssDNA by the A, B, C and D domains with increasing affinity as more domains are engaged^5^,^6^,^11^,^14^,^15^. The binding of ssDNA initially involves 70A and 70B and corresponds to excluded site size of 8–10 nucleotides (nt). This extends to a 20–30 nt binding mode when RPA is fully engaged^16^,^17^,^18^,^19^. A poorly characterized intermediate mode was proposed from previous experiments using fluorescence titration, isothermal titration calorimetry, and sedimentation that was assumed to engage the A, B and C domains^20^,^21^, but no direct evidence for this mode was found in X-ray scattering studies of ssDNA binding. The RPA70C domain in the RPA70 subunit contains a zinc-binding motif with the zinc ion bound to four cysteines^3^,^22^. The zinc-binding motif participates in the interaction with DNA^23^,^24^ and has been shown to be required for normal DNA replication^10^,^11^,^12^,^13^,^25^.

As a scaffold for recruitment of many downstream factors such as ATR and Rad51^9^,^23^, the stability and conformation of the RPA-ssDNA complex have to be tightly regulated. RPA has been reported to possess a high affinity, low cooperative binding to short ssDNA fragments with a dissociation constant (*K*_d_) ~50 nM^11^,^26^,^27^. The conformations of RPA-ssDNA complexes have also been studied using electron microscopy (EM) and atomic force microscopy (AFM) imaging, which reveal highly diverse conformations from amorphous random condensates to some extended filamentous like complexes^28^,^29^,^30^. While the causes of the highly diverse conformations of RPA-ssDNA complexes are not clear, they may depend on imaging procedures such as the negative staining in EM imaging and surface-sample interactions in AFM imaging. The amorphous random RPA-ssDNA conformations raise a question regarding how they can be recognized by downstream factors for recruitment.

Mechanical force has been increasingly recognized as a critical physiological factor involved in a variety of biological processes. In the nucleus, forces are produced during their actions on DNA of many active cellular machines such as DNA and RNA polymerase^31^,^32^. Since chromosomes have regions that are physically attached to the nuclear membrane, forces produced by actomyosin contraction of the cytoskeleton can be propagated to chromosomes through the nuclear membrane^33^,^34^. In addition, due to the physical attachment between the chromosome and nuclear membrane, forces are also produced passively by chromosomal packaging. As protein-DNA interaction typically involves a binding energy of several *k*_B_T and nm range interaction distances, forces of a few pN are expected to exist on chromosomal DNA due to DNA packaging.

As DNA polymerases are able to generate forces up to ~ 30 pN^31^,^32^, ssDNA produced during DNA replication is likely subject to force. During homologous recombination repair of double stranded DNA breaks (DSB), the two broken ends of DNA are tethered^35^ and recent *in vivo* dynamic imaging reveal that the ends move over large distance in a directed manner during the homologous search process^36^,^37^. These results imply that the DNA ends are likely subject to tension due to the physical tethering constraint. Nevertheless, the potential effects of force on the interaction between ssDNA and its processing factors are still largely unknown. In our research, we sought to elucidate how force regulates the conformation of RPA-bound ssDNA, which may provide novel insights into the regulation of the interactions between RPA, ssDNA and other cellular factors.

A truncated RPA construct, termed RPA DNA binding core (RPA-DBC), was used in this study. The RPA-DBC (RPA70_181-616_/RPA32_43-171_/RPA14) includes all of the linkers and domains (70A, 70B, 70C, 32D) that are involved in binding ssDNA. The deleted RPA70N and RPA32C are protein recruitment domains and the disordered N-terminus of the RPA32C subunit is a regulatory domain, none of which play a role in ssDNA binding. The full ssDNA binding apparatus of RPA and its binding properties are solely associated with the RPA-DBC, as reported previously^12^. In addition, the crystal structure and ssDNA binding activities of RPA-DBC have been previously characterized^12^,^38^.

By using a combination of AFM imaging and single-molecule stretching techniques, we report that in the absence of force RPA-bound ssDNA forms amorphous condensates, which is consistent with some previous imaging studies^30^. However, in the presence of a few pN forces, we found that RPA binding resulted in an extended ssDNA conformation with an increased effective bending rigidity of ssDNA. The effects of monovalent and divalent ions including sodium, magnesium, manganese, and zinc on RPA-ssDNA complex were analyzed by the mechanical response of RPA coated ssDNA. An interesting non-monotonic dependence of the binding affinity on the sodium concentration was revealed. In addition, a unique state was formed in the presence of micromolar zinc, one that results in a highly stable nucleoprotein complex that has not been reported in previous studies.

## Results

### RPA-DBC induces amorphous ssDNA aggregation in the absence of force

We used AFM imaging to visualize the conformations of RPA-bound ssDNA in the absence of force. Previously RPA-bound ssDNA has been imaged using EM and AFM, which revealed highly diverse conformations^28^,^29^,^30^. In these studies, negative staining or special multivalent cation dependent deposition methods were used to enhance the imaging contrast, which might be the potential causes of the observed diversity. To obtain the conformational information in the absence of force, we sought to use another deposition method that has been demonstrated to introduce less perturbation to protein-DNA complexes. This method uses glutaraldehyde-coated mica surface to crosslink the protein-DNA complex formed in solution to the mica surface^39^, which has been demonstrated to have less perturbation to DNA-protein complexes compared with other deposition methods such as APTES-mica and freshly cleaved mica^39^,^40^. In this method, the glutaraldehyde molecules are covalently coated on the surface; therefore, they do not diffuse into solution and won't cause random crosslinks. The tradeoff of this method is a reduced imaging contrast^29^,^40^.

**Figure 1:**
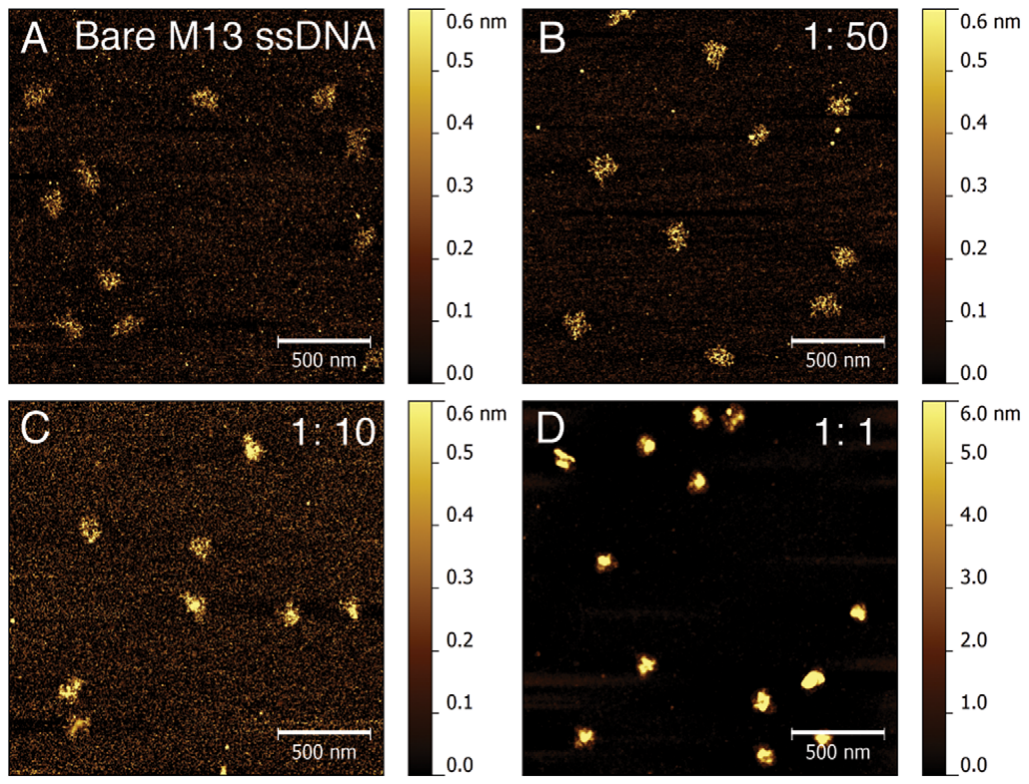
AFM images of RPA-bound ssDNA. (A). Randomly coiled naked M13 ssDNA. (B–D). RPA-bound ssDNA formed in different RPA to ssDNA stoichiometric ratios: 1:50 (B), 1:10 (C) and 1:1 (D). DNA concentrations in (A–D) were kept constant.

Because ssDNA is highly flexible and lacks regular structures, we chose a large ssDNA (M13, 7250 bp), which has a large random coil size for imaging. As shown in Fig. 1A, the naked M13 ssDNA molecules exhibit randomly coiled conformations, which is expected from the flexible ssDNA backbone characterized with a small bending persistence length of < 1 nm^41^. Fig. 1B–D show images of RPA-bound ssDNA formed in different RPA to ssDNA stoichiometries (1:50, 1:10 and 1:1), at a constant DNA concentration of 0.15 ng/μL. At a 1:1 ratio, the RPA-bound ssDNA appears as amorphous tight aggregates, in contrast to the randomly coiled loose conformations when complexes were formed at lower stoichiometries. To ensure that such amorphous aggregates are not caused by the glutaraldehyde-coated mica surface, we also performed similar experiments using other deposition/imaging methods including APTES-coated mica surface^42^ imaged in air, freshly cleaved mica deposited with magnesium solution imaged in air, and glutaraldehyde-coated mica surface imaged in-fluid, and obtained similar results (Supplementary Fig. S1).

### RPA induces extension elongation and stiffening of ssDNA molecules under force

The random amorphous conformations of the RPA-bound ssDNA, however, raise a question regarding how other cellular factors can access ssDNA in such dense aggregates. The RPA-ssDNA aggregates in Fig. 1(B–D) were formed in the absence of force. However, it has been known that forces in the physiological range of several pN can stretch the ssDNA from the randomly coiled conformation to a more extended conformation. We reason that RPA bound to an extended ssDNA conformation under force may lead to a more regular RPA-ssDNA complexes, suggesting force is a means for regulation of its accessibility.

**Figure 2:**
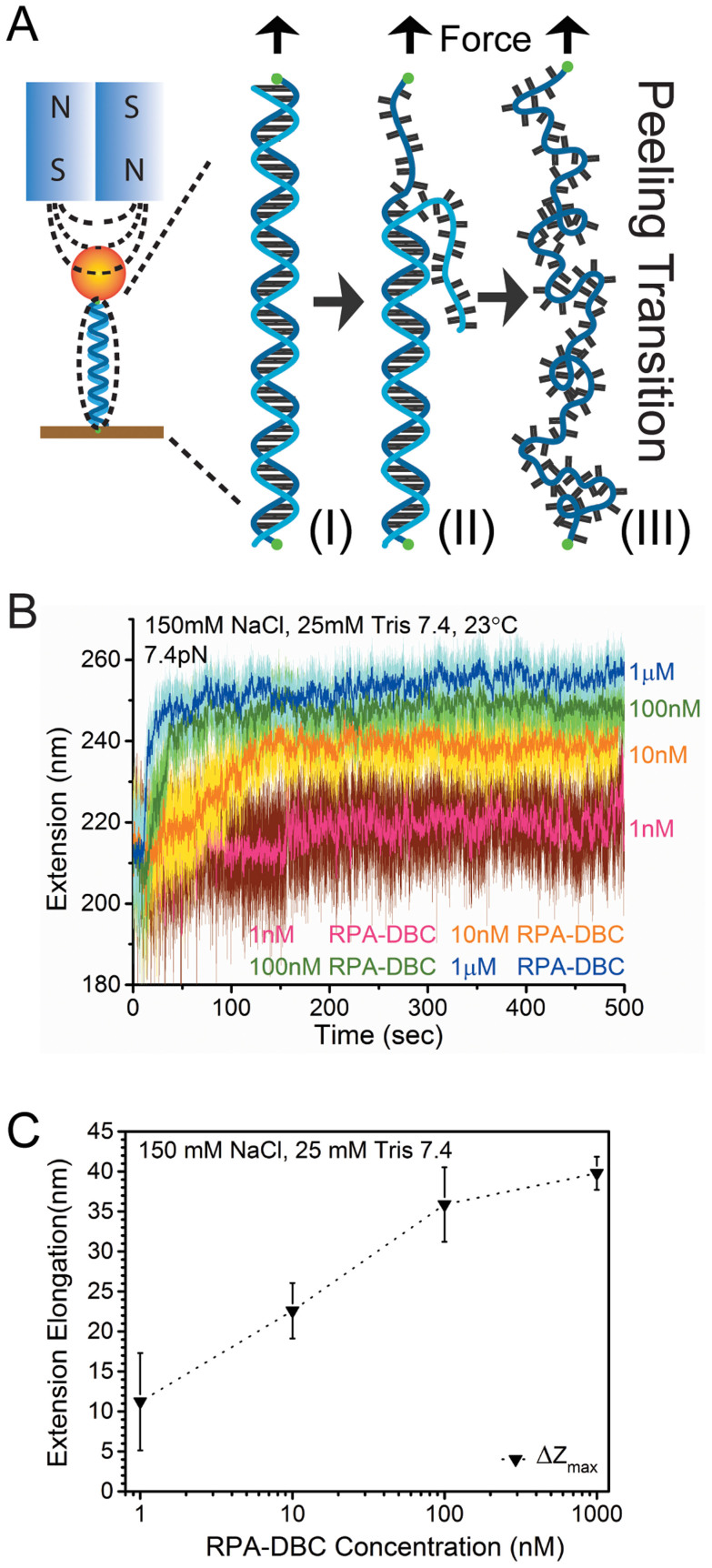
Concentration dependent dynamics of RPA's ssDNA binding. (A). A single 576 bp dsDNA is tethered between coverglass and paramagnetic bead surfaces through the two ends on the same DNA strand (I). By stretching the tethered DNA strand at ~ 65 pN, the untethered strand is peeled off (II), leaving an ssDNA strand under force (III). (B). Binding dynamics of RPA to ssDNA upon various protein concentrations from 1 nM–1 μM are introduced at a constant force of 7.4 pN smoothed with 30-points (0.3-second data) FFT filtering. (C). RPA concentration dependent DNA extension elongation Δz_max_ (tri-angles), which is the difference between the steady state extension (average extension in the last 100 seconds in Fig. 2B) and the naked ssDNA extension before introduction of RPA. Error bar for each symbol is the standard error (s.e.) from multiple (>3) independent measurements.

In order to investigate the force dependent conformations of RPA-bound ssDNA, we first measured the extension responses of a 576 nt ssDNA to RPA binding under constant forces. The 576 nt ssDNA is chosen because it is long enough to accommodate >20 RPA trimers, and short enough to achieve nm scale spatial resolution. Fig. 2B shows data obtained in a typical experiment done at 7.4 pN. The effect of RPA binding to ssDNA was observed from the resulting ssDNA extension change in real time after RPA was introduced at different concentrations from 1 nM to 1 μM on same ssDNA (Supplementary Fig. S2). At each RPA concentration, ssDNA extension increased with time after RPA was introduced until a steady state was reached. The time spent to reach the steady state is in a time scale from several seconds to ~100 seconds depending on RPA concentrations (Supplementary Fig. S3). These results reveal that RPA binding leads to a more extended ssDNA conformation, which depends on the protein concentration, as quantified by the total extension elongation until the steady state is reached (Fig. 2C). Similar observations were obtained for multiple independent experiments (> 7) (Supplementary Fig. S4). In contrast to previous kinetics studies in bulk on short ssDNA oligos (typically < 30 nt) that can only bind one RPA molecule^43^, this result provides observation of the real time binding dynamics of an array of RPA molecules to a single large ssDNA substrate under varying protein concentrations under force.

**Figure 3:**
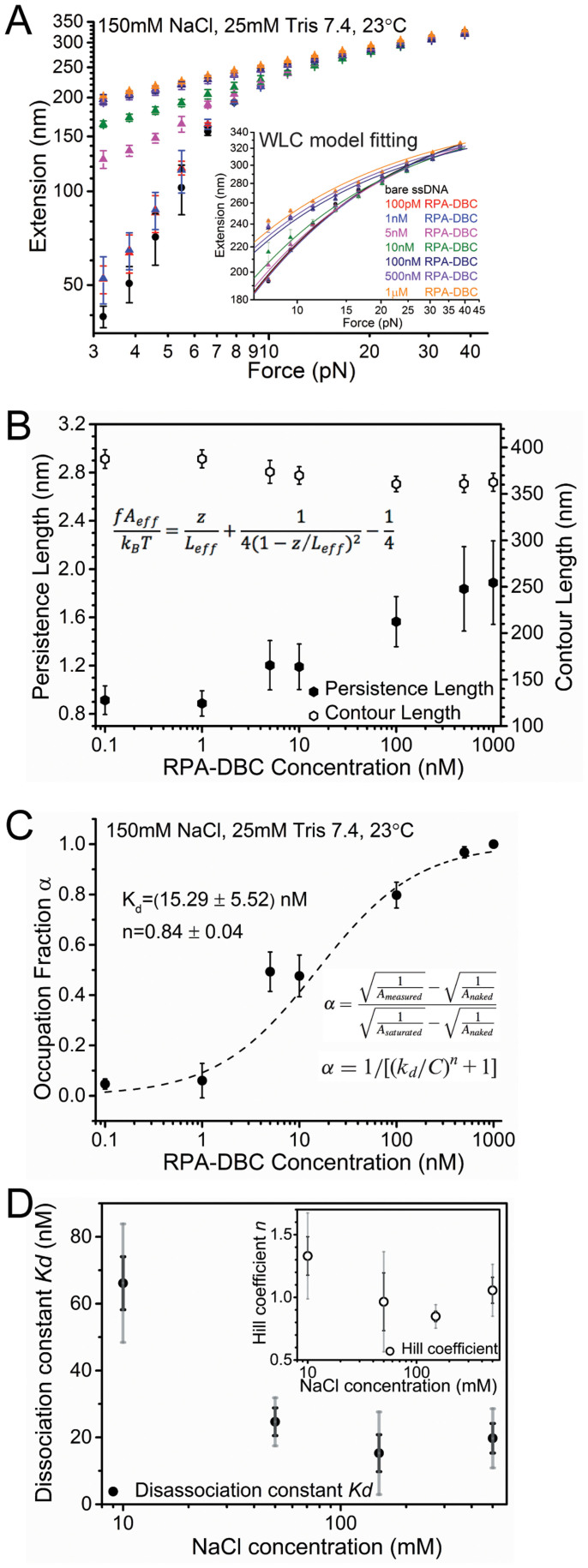
(A). Force-extension curves of RPA-bound ssDNA formed with varying RPA concentrations. Inset shows the fitting of experimental data in the force range of 7–45 pN by the Marko-Siggia formula (R^2^>96%), which gives the effective bending persistence length (A_eff_) and effective contour length (L_eff_) at each protein concentration (B). From the RPA dependent A_eff_, the RPA occupation fraction on ssDNA at different proteins concentrations are calculated and shown in (C). The error bars in (A) are standard deviation (s.d.) from multiple (>3) force-scans at each condition. The error bars in (B–C) are standard errors (s.e.) from multiple ssDNA tethers. The black dash line in (C) is average of Hill equation fitting of data obtained from multiple independent tethers (R^2^ > 91%). (D). Monovalent salt dependence of dissociation constant (solid circles) and Hill coefficient (Hollow circles, inset). The gray error bars indicate standard deviation (s.d.) and black error bars indicate standard errors (s.e.) from multiple (>7) ssDNA tethers.

The observation of ssDNA extension increase upon introduction of RPA under force is in contrast to the amorphous RPA-ssDNA aggregates formed in the absence of force, which should otherwise lead to significant extension reduction. That the RPA-bound ssDNA is longer than the naked ssDNA under the same force indicates that force changes the manner of RPA binding to the ssDNA. In order to gain further insights into the RPA-coated ssDNA under force, we carried out force-extension curve measurements to characterize its mechanical properties. At each RPA concentration, the force-extension curve could be reasonably fitted by the worm-like-chain (WLC) polymer model using the Marko-Siggia formula^44^ with an effective bending persistence length, *A*_eff_, and an effective contour length, *L*_eff_ (Fig. 3A &B).

We found that the fitted values of *L*_eff_ at different RPA concentrations are similar to that of naked ssDNA (difference is less than 6%). In contrast, the fitted value of *A*_eff_ increases as RPA concentration increases, and saturates at concentrations > 100 nM. As *A*_eff_ is a quantity reflecting the bending stiffness of a polymer, an increased *A*_eff_ indicates an overall stiffer ssDNA when it is bound by RPA. Our results indicate that RPA increases the apparent bending rigidity of DNA by 2–3 folds at saturation binding suggesting a moderate stiffening effect of RPA on ssDNA.

The increased *A*_eff_ is positively correlated with the fraction of RPA-bound ssDNA. As shown in previous publications^45^,^46^,^47^, the fraction of protein bound ssDNA at a given protein concentration, *α*(*c*), can be calculated from the concentration dependent *A*_eff_ (Fig. 3C). The binding affinity and cooperativity were analyzed by fitting *α*(*c*) with the Hill equation *α*(*c*) = 1/((*K*_d_/*c*)*^n^* + 1), which estimated a dissociation constant *K*_d_ of 15.29 ± 5.52 nM (mean ± standard error (s.e.)) and a Hill coefficient of *n* = 0.84 ± 0.04 (mean ± s.e.) in 150 mM KCl. This value of *K*_d_ is similar to that reported in previous biochemical measurements^12^,^48^. The Hill coefficient near one signifies low cooperativity in binding, which is also in agreement with previous studies^49^. These results indicate that force in this level does not change either the binding affinity or cooperativity significantly. We note that in such analysis, *K*_d_ was measured based on the change of the persistence length from the naked ssDNA to RPA-coated ssDNA until saturation in the same solution condition. Therefore, the measured *K*_d_ is not affected by the salt-dependence of the persistence length of ssDNA.

### Non-monotonic dependence of RPA-DBC binding to ssDNA on NaCl concentration

Sodium, potassium, magnesium, manganese and zinc are important cations that are known to provide oligonucleotides with distinct stabilities, biochemical properties and structures, and to greatly influence their processing by enzymes. To investigate their influence on RPA-bound ssDNA, magnetic tweezers experiments were repeated in the presence of monovalent (NaCl) and divalent (MgCl_2_, MnCl_2_, ZnCl_2_) cations.

Interestingly, the force-extension curve measurements and analysis at various NaCl concentrations in the range 10–500 mM (Supplementary Fig. S5) revealed a non-monotonic salt dependence of *K*_d_ (Fig. 3D), with the lowest value located at around 150 mM. Although the cause of the unexpected higher *K*_d_ at < 150 mM NaCl concentrations is not clear, we suspect that the RPA-trimer may have slight change in conformation or become unstable and therefore bind with lower affinity at low NaCl concentration. The higher *K*_d_ at > 150 mM NaCl concentration can be explained by electrostatic screening. In the range of NaCl concentrations tested, the Hill coefficient shows a negligible dependence on salt, with an average value near one (Fig. 3D, inset).

### Zinc drives RPA-coated ssDNA into a tightly wrapped, highly stiff conformation

As regard the effects of divalent ions, analysis of force-extension curves showed that the presence of magnesium and manganese have virtually no effect on the force responses of RPA-coated ssDNA after hours of incubation at low force (Supplementary Fig. S6). However, the addition of zinc was associated with remarkably large effects on the RPA-coated ssDNA.

**Figure 4:**
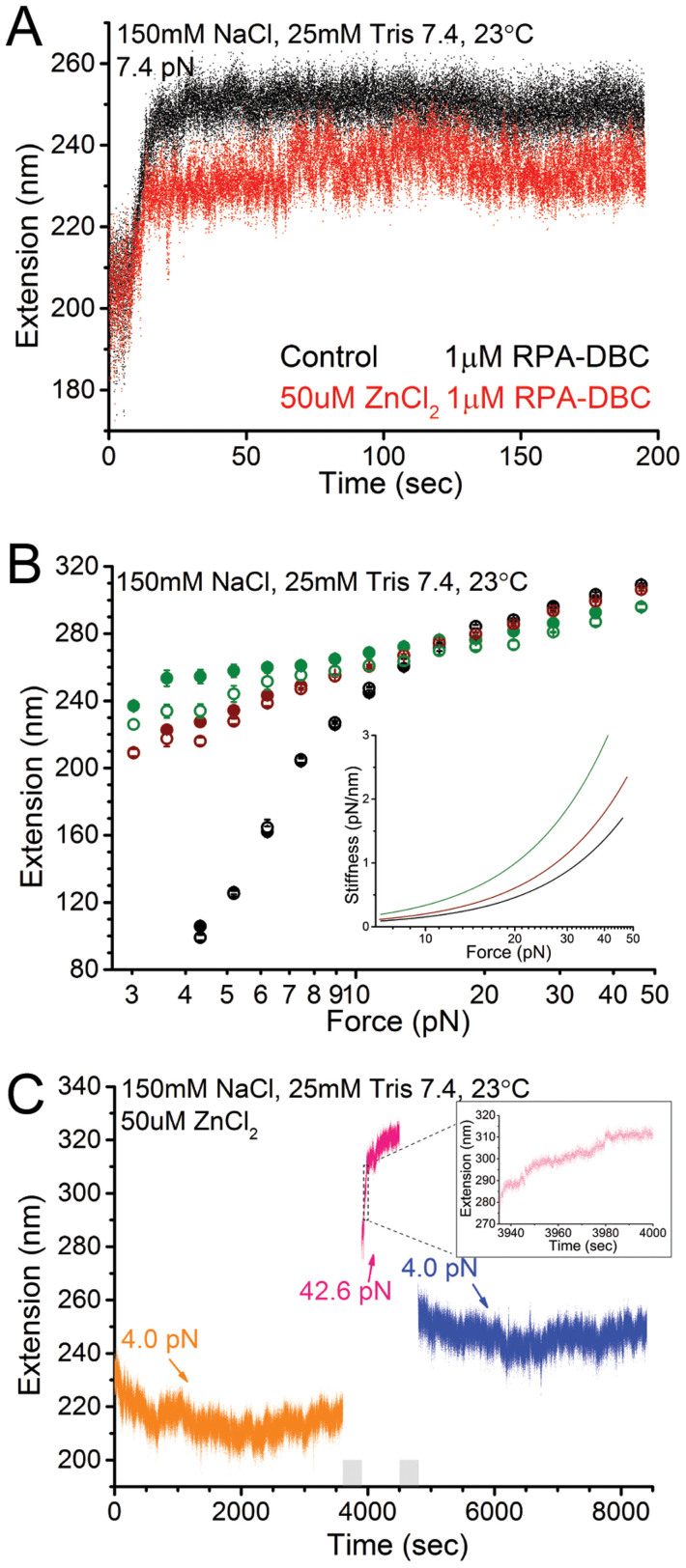
(A). Comparison of the binding dynamics of 1 μM RPA to ssDNA in the absence (black) and presence (red) of zinc under constant force of 7.4 pN, showing a different steady state extension. (B). Comparison of the steady state force-extension curves between zinc-free (wine) and zinc-induced (olive) RPA-bound ssDNA formed after saturation binding at 7.4 pN. Data indicated by solid and hollow symbols are obtained during force-decrease and force-increase scans, respectively. Force-extension curve of bare ssDNA (black) is plotted for comparison. Inset show the quantification of the stiffness of ssDNA and RPA-bound ssDNA. (C). Extension time trace of RPA-bound ssDNA at constant forces in the presence of zinc. Inset shows the zoom in of extension increase of the initial 70 s after force jumped from 4 pN to 43 pN.

Fig. 4A shows extension elongation time trace of ssDNA after solutions of 1 μM RPA were introduced at 7.4 pN in the presence of 50 μM ZnCl_2_. An initial rapid elongation followed by saturation binding kinetics was observed, with a relaxation time scale similar to that without zinc (Supplementary Fig. S7A). However, the extension change up to saturation, Δ*z*_max_, was shorter than that obtained in the absence of zinc at the same force (Supplementary Fig. S7B). After near saturation was reached at 7.4 pN, the force-extension curves were measured by decreasing force sequentially (force-decrease scan) followed by increasing force back (force-increase scan) through the same set of force values. At each force, the tether was held for 5 seconds to obtain the average extension. The resulting force-extension curves (Fig. 4B) obtained in the force-decrease scan and the subsequent force-increase scan show a small hysteresis, suggesting that the RPA-coated ssDNA was not at equilibrium, which was not seen in the absence of zinc over the same time scale.

The force-extension curves of RPA-coated ssDNA formed in zinc solution has a flatter profile compared to that formed without zinc, indicating an increased stretch stiffness *k* = *df*/*dz*, where *f* is force and *z* is extension. The effective stretch stiffness was calculated by the derivatives of the fitted WLC force-extension curves to the measured force-extension data at forces > 7 pN (Fig. 4B, inset). Note that the data obtained in the force-decrease scan in the presence of ZnCl_2_ were used for the fitting to minimize the influence from the hysteresis. The analysis shows that RPA increases the stretch stiffness, which is further enhanced by zinc.

Fig. 4B shows that the DNA extension recorded in the presence of ZnCl_2_ is slightly shorter than that in zinc free condition at forces up to 30 pN. This is also the case when ZnCl_2_ is mixed with MgCl_2_ and MnCl_2_ (Supplementary Fig. S9). These results suggest that in the presence of Zn^2+^, the ssDNA may wrap around RPA during scans at lower forces in the scanned force range. ssDNA wrapping is anticipated to be more prominent at lower forces, so this was tested by holding the same tether under a decreased force (4.0 pN) and observing the dynamic change of extension. As expected, the extension progressively decreased by ~20 nm (Fig. 4C, orange data), which took >30 min to reach a nearly steady state. In order to further understand how RPA-bound ssDNA responds to force, force was jumped from 4 pN to 43 pN and remained there for over 600 seconds (Fig. 4C, pink data). Immediately following the force jump, the extension increased with time at a speed of ~ 0.3 nm/s in the first 100 seconds, then reached a nearly steady state with a speed less than 0.02 nm/s (Fig. 4C, pink data). Zooming in to the first 70 seconds after jumping from 4 pN to 43 pN during which the extension quickly increased reveals that the extension increase is a gradual process without abrupt extension jumps (Fig. 4C, inset). At the final step, force was jumped back to 4 pN and the tether was held at the force for one hour. Over the holding time, the overall extension only reduced by ~5 nm (Fig. 4C, blue data) indicating that the structure formed at 43 pN is highly stable and does not relax to lower extensions as shown in orange data within our measurement timescales.

These results suggest that the presence of micromolar zinc causes RPA-coated ssDNA to rearrange its conformation into one that is stiff and more wrapped when it is incubated at the lower force, which can be unwrapped to some extent at increased forces. This is consistent with a picture of slow local rearrangement of the conformation as opposed to unlooping of juxtaposed remote DNA sites by RPA. The rearrangement into a new nearly steady extension upon force change typically takes minutes depending on the level of force, indicating a slow relaxation process. This result is in contrast to the case in the absence of zinc, where such slow conformational relaxation processes were not observed.

Overall, taking together the results in Fig. 3 and Fig. 4, an unexpected zinc dependence of RPA-coated ssDNA is revealed. Without excess zinc, RPA binding to ssDNA quickly reaches equilibrium, and the resulting force-responses of RPA-coated ssDNA can be described by a simple worm-like chain polymer model with a RPA concentration dependent effective bending persistence length. In sharp contrast, in the presence of micromolar zinc, RPA binds to ssDNA but does not reach equilibrium even over long experimental time scales. The conformation of RPA-coated ssDNA in excess zinc can be deformed by force. However, upon force change, relaxation of the RPA-coated ssDNA to a new conformation takes a very long time. The stiffness measurement before significant force-dependent relaxation takes place reveals that the zinc induced RPA-coated ssDNA is a highly stiff structure compared to that without zinc.

Furthermore, formation of such highly stiff RPA-coated ssDNA seems to be specific to zinc ion concentrations >10 μM, as it was not observed in experiments with other divalent ions such as manganese and magnesium or at lower zinc concentrations. Control experiments of stretching ssDNA in zinc solution without RPA-DBC have shown that this zinc effect is not caused by zinc mediated ssDNA rearrangement (Supplementary Fig. S8).

## Discussion

In this study, we have investigated the effects of physiological range of force on RPA binding to ssDNA and the micromechanics of the resulting RPA coated ssDNA. Our results have revealed several highly interesting mechanochemical properties of RPA-coated ssDNA.

Previously, binding of one RPA molecule was extensively studied using short ssDNA substrates (typically <30 nt) in the absence of force^13^,^20^,^50^, which provides information on binding rates and stability of a single RPA-bound ssDNA complex as well as RPA diffusion on ssDNA. Binding of multiple RPA molecules to larger ssDNA substrates in the absence of force has been investigated by the gel mobility shift assay^49^, electron microscopy^28^, and DNA curtain imaging^51^. To our knowledge, there is only one study of RPA binding to DNA molecules under force using single molecule force manipulation technology, which however focused on the effects of RPA dependent unwinding of double-stranded DNA rather than understanding the effects of force on RPA binding to ssDNA^52^. Our work extends many important results obtained from these previous studies by providing information regard how force regulates RPA binding to ssDNA and the mechanochemical properties of the resulting nucleoprotein complex.

Our AFM imaging revealed that RPA can induce ssDNA condensation into amorphous aggregates in the absence of force as shown in our AFM and some previous EM imaging studies^30^. In contrast to RPA-bound ssDNA aggregates, a previous AFM imaging experiment reported filamentous nucleoprotein structure of RPA-coated ssDNA, where spermidine solution was used to assist deposition of the RPA-bound ssDNA onto the negatively charged freshly cleaved mica surface. Among several deposition methods tested, we were able to reproduce such filamentous RPA-ssDNA complexes only when we deposited the RPA-bound ssDNA using spermidine solution (Supplementary Fig. S1). Therefore, it seems to us that such extended filamentous nucleoprotein structures are likely dependent on the presence of spermidine.

**Figure 5:**
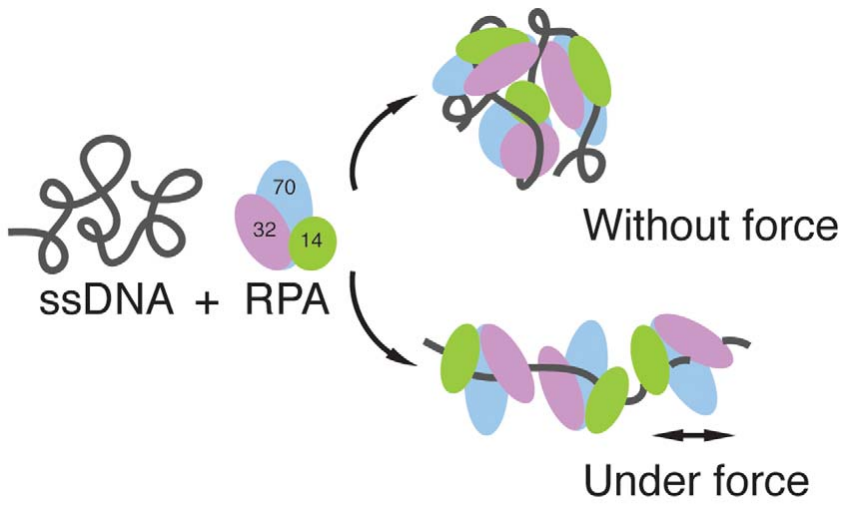
Schematics of a model that describes the effect of force on the conformation of RPA-coated ssDNA. In the absence of force, RPA ssDNA binding domains bind distant ssDNA sites, stabilizing condensed ssDNA. In contrast, in the presence of force, RPA binds to the force extended ssDNA conformation, resulting in an ordered extended conformation with increased bending rigidity.

We show that when force is applied to ssDNA, RPA binding results in an extended conformation indicating an apparently increased DNA bending rigidity. This is in sharp contrast to RPA induced ssDNA condensation into amorphous aggregates in the absence of force as shown in our AFM and previous EM imaging studies. These results suggest that force can regulate the binding modes of RPA, switching it from causing disordered ssDNA condensation to a much more extended conformation (see sketch Fig. 5). As ssDNA produced during replication and DNA damage repair is likely subject to force, such extended RPA-bound ssDNA conformations may be physiologically relevant: an extended RPA-bound ssDNA has an obvious advantage in regulation of the ssDNA accessibility by other cellular factors. The effect of force can be explained by two possible factors, one is to remove the secondary structures on ssDNA that facilitates RPA binding, and the other is to prepare a stretched ssDNA conformation that may guide the binding of RPA into a final extended nucleoprotein complex.

Our analysis of the RPA concentration dependent effective bending persistence length shows that RPA binding to ssDNA has a low cooperativity and a dissociation constant around 10 nM, which is consistent with previous biochemical measurements in the absence of force^13^,^49^. This result suggests that force does not significantly affect the binding affinity and the cooperativity. The fitted effective contour length of RPA-coated ssDNA in various RPA concentrations remains similar to that of naked ssDNA over the force range tested, indicating that RPA binding does not wrap ssDNA that should result in reduced amount of free ssDNA, in contrast to the bacterial single-stranded DNA binding protein (SSB) known to wrap ssDNA^53^,^54^,^55^.

An unexpected non-monotonic dependence of *K*_d_ of RPA on NaCl concentration was observed. In the range 150–500 mM, *K*_d_ increases as NaCl concentration increases, consistent with the expectation from basic principles of electrostatic screening. In the range of 10–150 mM, *K*_d_ decreases as NaCl concentration increases, in contrast to predictions based solely on electrostatic screening. Low ionic strength may induce a different conformation in RPA-trimer and/or alter its DNA binding properties. An alternative explanation is that RPA may be destabilized in low monovalent salt concentrations in a manner that decreases its ability to bind the DNA substrate. This complex NaCl concentration dependence of *K*_d_ signifies that, besides affecting electrostatic interaction between RPA and ssDNA, NaCl may also change the RPA trimer conformation. Further investigations are needed to fully understand this result.

Previously it was reported that RPA is able to unwind dsDNA at low mono- or divalent salt concentration (< 120 mM NaCl)^52^. This result is not in contrast to our observation of increased *K*_d_ of RPA-DBC to ssDNA at such low salt concentrations, since the RPA induced dsDNA unwinding depends on not only RPA-ssDNA binding affinity but also DNA hybridization, with the latter as the predominant factor (Supplementary Discussion S1). As a result, lowering salt concentration always facilitates RPA binding to melted bubbles in dsDNA, even when the RPA-ssDNA binding affinity decreases at the lowered salt concentration.

Interestingly, we show that micromolar concentration of zinc ions drives the formation of a distinct, highly stiff RPA-coated ssDNA state, indicating a different mode of interaction of RPA with ssDNA in the presence of zinc under force. Together with our results showing that manganese and magnesium do not induce a similar effect; these data reveal this is a zinc-specific phenomenon. Moreover, this zinc-induced effect can be undone by addition of EDTA (Supplementary Fig. S10). As the zinc-binding motifs typically have nanomolar range dissociation constants^56^, this zinc-induced effect is unlikely related to the zinc-binding motif in RPA70. We emphasize that the observed zinc-induced effect is not caused by zinc-induced self-aggregation of RPA (Supplementary Fig. S11), or zinc-induced ssDNA reorganization (Supplementary Fig. S8), or loss of function of RPA over long time scale of experiments in room temperature (Supplementary Fig. S12).

The concentration range of ZnCl_2_ (>10 μM) at which such phenomenon was observed is consistent with the overall zinc concentration in cells, although it is considerably greater than the nM concentration of free zinc^57^. However, it is increasingly apparent that the concentration of free zinc can fluctuate, including under conditions where cells are under stress^58^, which suggests there might in fact be a physiological role, albeit rare, for this extraordinary stable, zinc-induced RPA-ssDNA state.

## Methods

### DNA and protein

A 576 bp double stranded (ds) DNA was generated by DreamTaq polymerase (Fermentas) catalyzed PCR. A 576 nt single stranded (ss) DNA was created using a previously published protocol where a tethered 576bp dsDNA was melted through a force-induced DNA strand-separation transition^59^,^60^ (Fig. 1A). The human RPA-DBC (RPA70_181-616_/RPA32_43-171_/RPA14) was purified as previously described^12^. RPA-DBC ssDNA binding assays were performed at 23 °C in a buffered solution containing 10–500 mM NaCl, 25 mM Tris pH 7.4 with or without ZnCl_2_, MnCl_2_, or MgCl_2_.

### Atomic force microscope imaging

Circular M13mp18 ssDNA (NEB) was mixed with RPA-DBC in nucleotide: protein ratio denoted in the figure legend. Glutaraldehyde-coated mica surface was used to crosslink RPA-DBC coated ssDNA complex onto mica surface for imaging. On such glutaraldehyde-coated mica surface, the glutaraldehyde molecules are covalently coated on the surface. Any free glutaraldehyde molecules were removed by rinsing the surface with distilled water to avoid glutaraldehyde induced inter-protein crosslinking in solution. Detailed surface preparation procedures can be found in previous publications^39^,^40^. It has been reported that the glutaraldehyde-coated mica surface has a minimal perturbation to the conformations of nucleoprotein complexes compared to freshly cleaved mica and APTES-coated mica^39^,^40^. Furthermore, as the immobilization of nucleoprotein complexes on glutaraldehyde-coated surface does not depend on specific solution requirements, we were able to image RPA-ssDNA in different solution conditions. In our experiments, the protein and DNA mixture was incubated in the buffer condition of 150 mM NaCl, 25 mM Tris 7.4, 23 °C for 30 mins before deposition followed by deposition on glutaraldyhyde mica for 15 mins. After that the sample was gently washed by deionized water and dried using clean nitrogen gas. The image is scanned using Bruker's Fastscan AFM under tapping mode.

### Magnetic tweezers setup and extension measurement

In order to investigate protein and DNA interaction activities at single DNA level, a high-force magnetic tweezers apparatus with a spatial resolution of ~2 nm and temporal resolution of 100 Hz was built^61^. A 576 bp double stranded (ds) DNA labeled with biotin and thiol on 5′ and 3′ of the same strand was specifically tethered between a coverslip and paramagnetic bead (Dynabeads M280 streptavidin, Invitrogen), respectively. The single DNA tether was verified and validated according to force induced overstretching at around 65 pN marked with 1.7 times elongation. Beads rotation caused extension measurement errors can be eliminated according to a previous protocol^53^,^62^. First, force-extension curve of a dsDNA were measured between 4 pN -40 pN as reference. At the same time, a theoretical dsDNA force-extension curve based on worm-like-chain polymer model^44^ (Eq. 1) is calculated at the same set of forces. Then, force-extension curves of the resulting ssDNA were measured as the extension difference between the ssDNA and the original dsDNA before melting at the same set of forces plus the theoretical dsDNA force-extension curve. 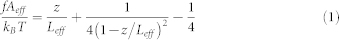
The worm-like-chain polymer model indicating that the relationship between DNA end-to-end distance (*z*) and applied force (*f*) is dependent on DNA contour length (*L*_eff_), persistence length (*A*_eff_).

### Quantification of DNA stiffness, occupation fraction and binding kinetics

Stretch stiffness is calculated by the first order derivative of force to extension (*d*f/*d*z) for the fitted worm-like chain force-extension curves.

The occupation fraction was correlated with variation of effective persistence length of bare ssDNA and protein coated ssDNA, which can be calculated according to (Eq. 2).
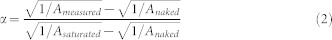
*A*_naked_, *A*_measured_ and *A*_saturated_ represent the effective persistence length of bare ssDNA, protein coated ssDNA at a given protein concentration and protein-coated ssDNA above the protein saturation concentration, which shows that a higher protein binding occupation fraction is marked with an increased persistence length of protein coated ssDNA complex. Since ssDNA occupational fraction has a certain value under a given protein concentration, protein's binding kinetics can be quantified by fitting occupational fraction (α) and protein concentration (c) according to Hill equation (Eq. 3). 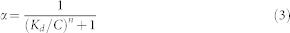


## Author Contributions

J.Y. and W.J.C. conceived the research. J.C., S.L. and J.Y. designed the experiments. J.C., S.L. and A.B. performed the experiments. J.C. and S.L. performed data analysis. J.C., S.L. and J.Y. interpreted the data. J.C., S.L., W.J.C. and J.Y. wrote the paper. The authors declare no conflict of interest.

## Supplementary Material

Supplementary Information Supplementary information for Mechanochemical regulations of RPA's binding to ssDNA
