# Supplementary material for: Mechanochemical regulations of RPA's binding to ssDNA
Source: Sci Rep. 2015 Mar 19;5:9296. doi: 10.1038/srep09296 (PMC4365408; doi:10.1038/srep09296)
Supplement: Supplementary Information — for Mechanochemical regulations of RPA's binding to ssDNA [file srep09296-s1.pdf]

Supplementary information for

Mechanochemical regulations of RPA's binding to ssDNA

Jin Chen, Shimin Le, Anindita Basu, Walter J. Chazin, Jie Yan

Supplementary Discussion S1

In this study, we report the non-monotonic dependence of RPA binding affinity to ssDNA on NaCl concentration with the lowest K_d value located at around 150 mM. Previously, de Vlaminck et al. studied RPA binding to ssDNA inside dsDNA at low force. They reported that the stability of the RPA bound in melted bubbles inside the dsDNA increases as salt concentration decreases in the range of 30 mM to 120 mM of NaCl. In that study, the RPA induced dsDNA unwinding depends on not only RPA-ssDNA binding affinity but also the DNA hybridization, with the latter as the predominant factor. As a result, lowering salt always facilitates RPA binding to melted bubbles in dsDNA, even when the RPA-ssDNA binding affinity decreases at lower salt concentrations. The details are explained below.

Lower salt has a strong destabilizing effect on the stability of DNA duplexes. Using the well understood salt dependent base pair stability (Santa Lucia, PNAS, 1999), decreasing from 150 mM to 10 mM NaCl should cause a decrease of ~ 0.8 k_BT per base pair, or ~24 k_BT considering that one RPA engages ~ 30 nt of ssDNA. This duplex destabilizing effect can facilitate RPA binding by dramatically reducing the free energy cost to form a DNA bubble for binding by an RPA molecule. On the other hand, our result shows that k_D of the RPA-ssDNA complex increases about three folds when salt concentration decreases from 150 mM to 10 mM. This corresponds to a decreased ssDNA binding free energy by ~ 2.7 k_BT per RPA, which is about one order of magnitude smaller than the decreased base pair energy for 30 bp DNA that can be bound by one RPA.

This free energy cost estimation suggests that the low salt-induced base pair destabilization is a predominant factor that governs the salt dependent RPA binding to dsDNA bubbles. Lowering salt therefore always facilitates RPA binding to melted bubbles in dsDNA, even when the RPA-ssDNA binding affinity decreases at lower salt concentrations. As such, our results obtained from RPA binding to ssDNA are not contradicting to the previous studies of RPA-dsDNA binding by de Vlaminck et al.

Supplementary Figures:

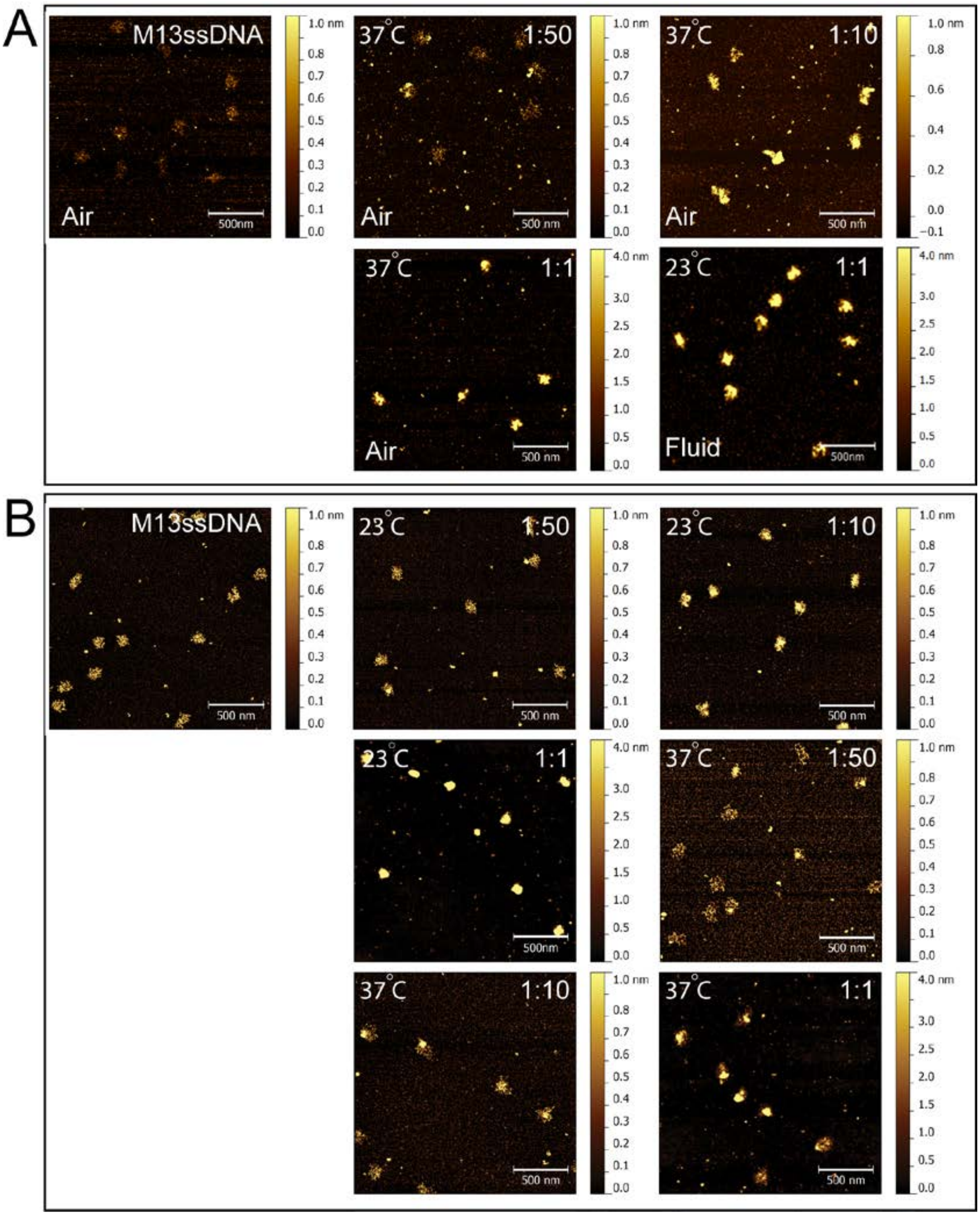

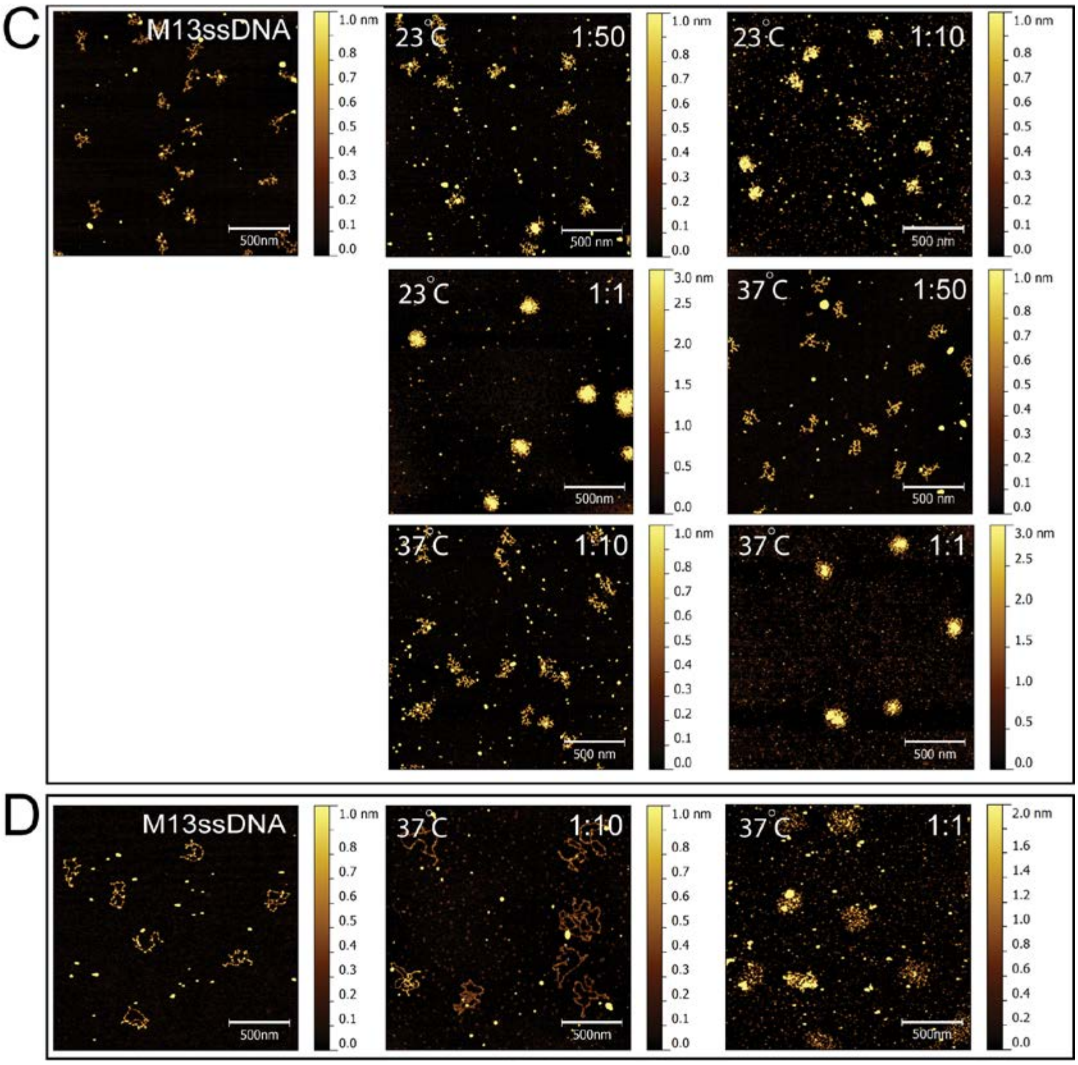

Supplementary Figure S1. AFM images of M13 ssDNA and M13 ssDNA incubated with varying concentration of RPA with different mica surfaces including: glutaraldehyde coated mica surface imaged in air/fluid (A), APTES coated mica surface imaged in air (B), freshly cleaved mica deposited with magnesium solution imaged in air (C), freshly cleaved mica deposited with spermidine solution imaged in air (D). The RPA-to-nucleotide ratio, temperature, and scale bars are indicated in the respective figure panels.

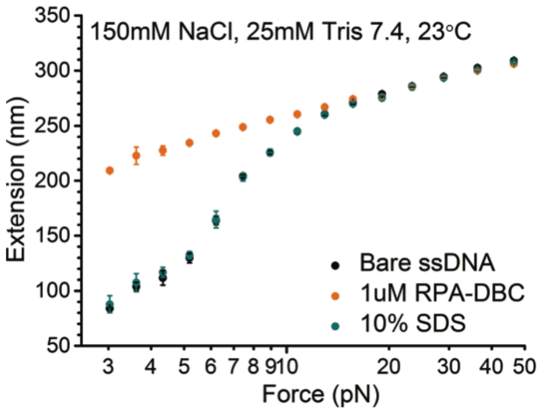

Supplementary Figure S2. The RPA bound on ssDNA can be removed by SDS wash. Force-extension curves of a fresh 576 nt ssDNA (black), the same DNA after incubation with 1 μM RPA (orange), the same DNA after RPA was washed with 10% SDS solution (dark cyan). Error bars are s.d. of obtained from multiple (>3) force cycles including force increase and force decrease. The results indicate that the bound RPA could be washed; therefore the same DNA could be repeatedly used to multiple experiments at different protein concentrations and solution conditions.

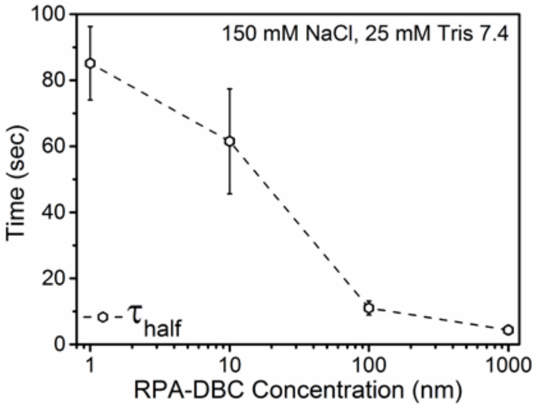

Supplementary Figure S3. RPA concentration dependent DNA extension half time. τ_{half} is defined when the extension elongation is half to the maximal change, which reflect the time required for steady state of RPA coated ssDNA. Error bar for each symbol is the standard error (s.e.) from multiple (>3) independent measurements.

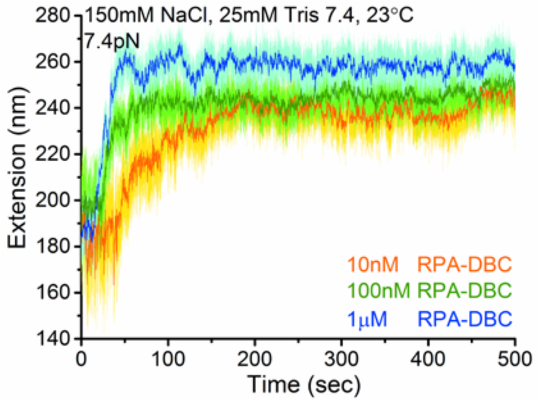

Supplementary Figure S4. Concentration dependent dynamics of RPA's ssDNA binding. Binding dynamics of RPA to ssDNA upon various RPA concentrations from 10 nM - 1 μM were introduced at a constant force of ~ 7.4 pN on an ssDNA tether independent from the one used in Fig. 2B in the main text. Colored solid and dot lines indicate the initial extension of ssDNA before binding and the averaged steady state extensions after binding, respectively. The results are essentially the same as those in Fig. 2B. Similar results were obtained in > 3 independent experiments.

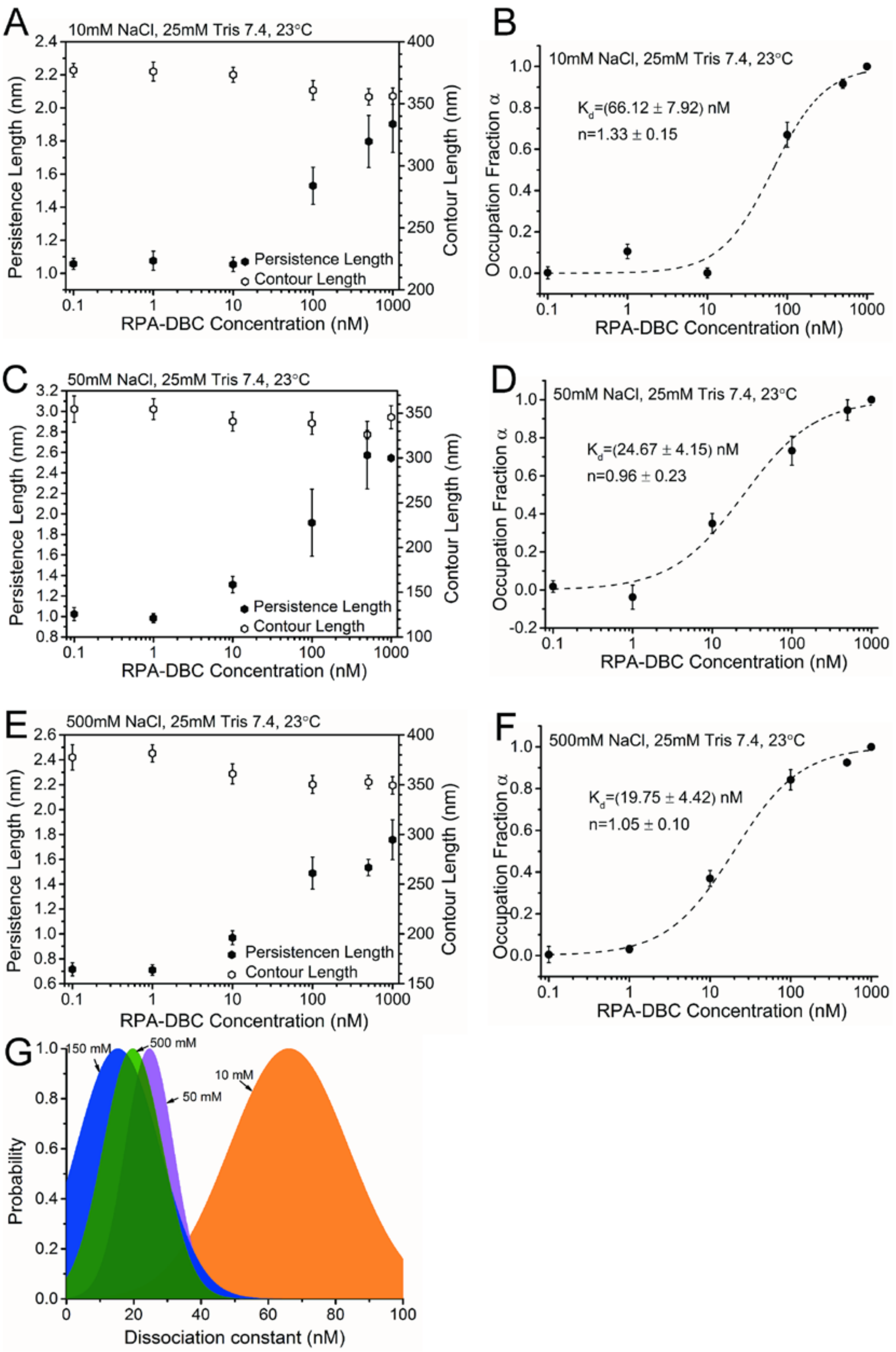

Supplementary Figure S5. Hill equation fitting of RPA's ssDNA binding in NaCl concentration from 10 mM to 500 mM. RPA concentration dependent effective bending persistence length, A_{eff} and effective contour length L_{eff} of RPA coated ssDNA were fitted to the WLC model, and resulting occupancy factors were calculated at 10 mM (A-B), 50 mM (C-D) and 500 mM (E-F) NaCl and 25 mM Tris (pH 7.4). The symbols and the error bars are the average value and s.e from multiple independent experiments (> 3). The black dash lines in (B,D, & F) are average of Hill equation fitting of multiply independent tether (R^2 > 91%) with dissociation coefficient and Hill coefficient as insert legends. (G) At each salt concentration,

the uncertainty of the measured dissociation constant is represented by Gaussian distribution, plotted with the average and standard deviation estimated from experiments. The resulting profiles are distinguishable from one to another, particularly between 10 mM and higher salt concentrations.

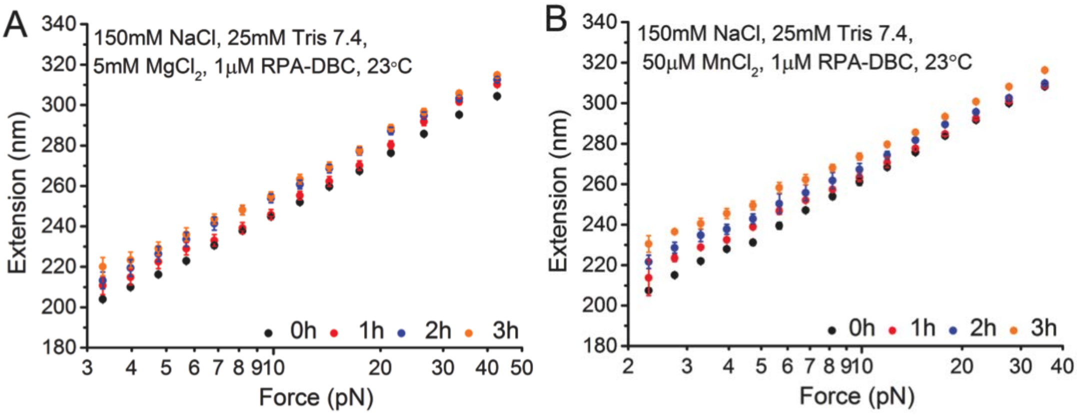

Supplementary Figure S6. Effects of magnesium or manganese on the force responses of RPA coated ssDNA. The force-extension curves of single ssDNA incubated with 1 μM RPA in buffer solution containing 5 mM $MgCl_2$ (A) or 50 μM $MnCl_2$ (B) measured after different incubation times at 2.6 pN: right after protein introduction (black), after 1 h (red), after 2 h (blue), and after 3 h (orange) of incubation. The force extension curves are similar to the one without magnesium or manganese (Fig. 3A).

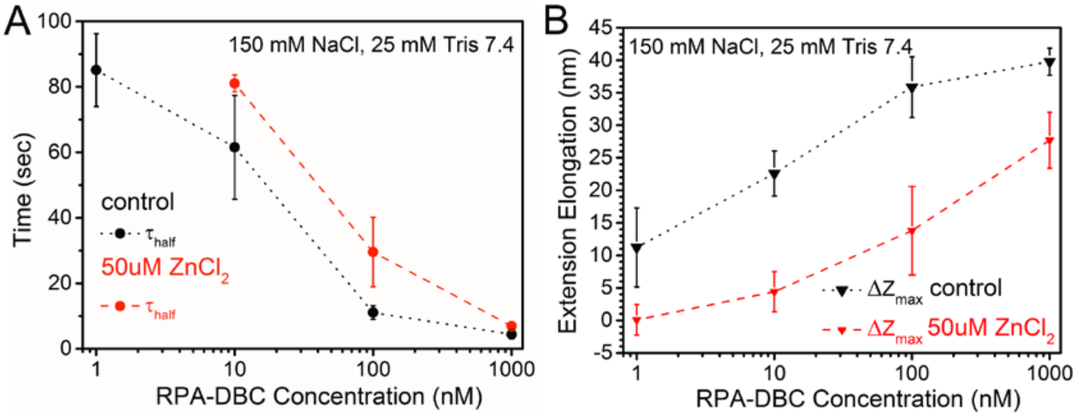

Supplementary Figure S7. Effect of zinc on the dynamics of RPA's ssDNA binding. (A). RPA concentration dependent DNA extension elongation relaxation time at zinc free condition (black, circle) and with 50 μM $ZnCl_2$ (red, circle). Hollow circles indicate half relaxation time τ_{half}. (B). RPA concentration dependent (1 nM - 1 μM) DNA extension elongation Δz_{max} at zinc free condition (black, triangle) and with 50 μM $ZnCl_2$ (red, triangle) after near steady state have been reached at 7.4 pN. Error bars are the standard errors (s.e.) estimated from multiple (>3) independent measurements.

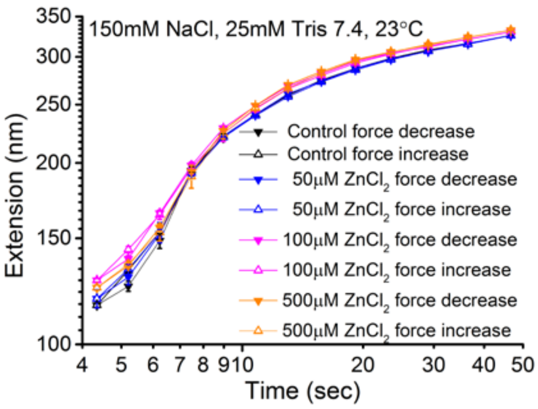

Supplementary Figure S8. Effect of zinc on bare ssDNA. The force extension force decrease (solid) and force increase (hollow) curves of bare ssDNA in zinc free (black), 50 μM $ZnCl_2$ (blue), 100 μM $ZnCl_2$ (magenta) and 500 μM $ZnCl_2$ (orange) conditions, which show zinc has zero effect on ssDNA extension behaviors. In addition, over 1h incubation of ssDNA under low force in zinc buffer also had no effect on its force responses (data not shown).

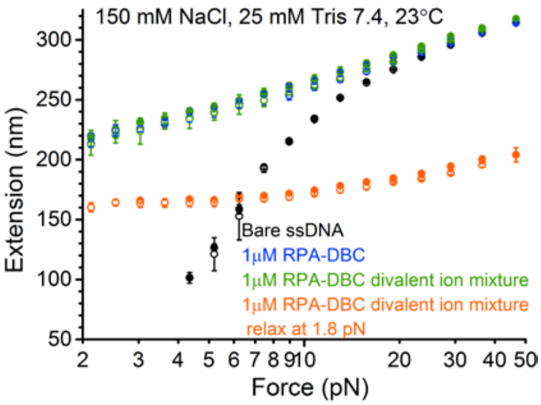

Supplementary Figure S9. Force response of RPA coated ssDNA with a mixture of 50 μM zinc, 5 mM magnesium and 50 μM manganese. Force-decrease (solid symbols) and force-increase (hollow symbols) curves of naked ssDNA (black), right after introduction of RPA without zinc (blue), right after introduction of RPA with the mixture of the divalent ions (olive), after incubation at 1.8 pN for two hour (orange symbols). The result shows that the zinc introduced slow rearrangement of the conformation of RPA coated ssDNA into a more compact rigid one.

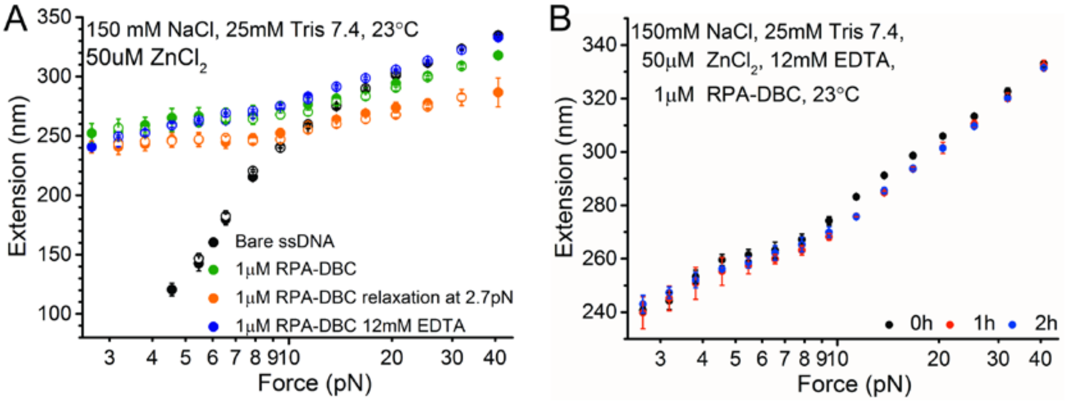

Supplementary Figure S10. Force responses of RPA coated ssDNA after addition of EDTA with zinc. (A). Force-decrease (solid symbols) and force-increase (hollow symbols) force-extension curves of naked ssDNA (black symbols), right after introduction of RPA with zinc while without EDTA (olive symbols), after incubation at 2.6 pN for one hour (orange

symbols), and immediately after introduction of RPA with zinc and EDTA (navy symbols). (B). Force extension curves obtained in mixture of 12 mM EDTA, 50 M $ZnCl_2$ and 1 M RPA right after the introduction (black), after 1 h (red), 2 h (blue) and 3 h (orange) incubation at 3.8 pN. These results show that EDTA can remove the influence of zinc on the force response of the RPA coated ssDNA.

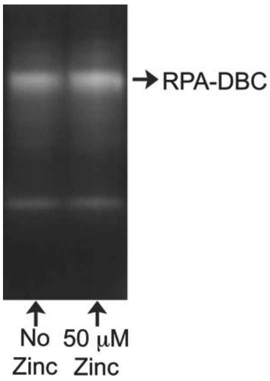

Supplementary Figure S11. Native polyacrylamide gel electrophoresis with 8% acrylamide shows 2 μg RPA in zinc free condition (left lane) and 50 μM $ZnCl_2$ (right lane). The two lanes appear basically same and show no sign of protein self-aggregation.

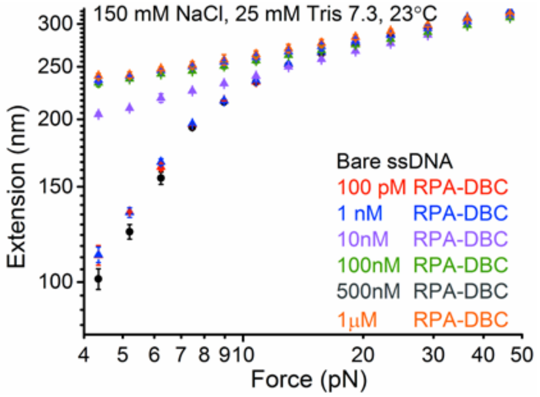

Supplementary Figure S12. Force-extension curves of ssDNA coated with varying concentration of RPA after the RPA is placed at room temperature for 5h are is similar to those measured for freshly thawed RPA in Fig. 2A, indicating that the RPA ssDNA binding property remain unchanged over such experimental time scale.
